# Nobiletin resolves left ventricular and renal changes in 2K-1C hypertensive rats

**DOI:** 10.1038/s41598-022-13513-6

**Published:** 2022-06-03

**Authors:** Metee Iampanichakul, Anuson Poasakate, Prapassorn Potue, Siwayu Rattanakanokchai, Putcharawipa Maneesai, Parichat Prachaney, Wannapa Settheetham-Ishida, Poungrat Pakdeechote

**Affiliations:** 1 Department of Physiology, Faculty of Medicine, Khon Kaen University, Khon Kaen, 40002 Thailand; 2 Faculty of Veterinary Medicine, Khon Kaen University, Khon Kaen, 40002 Thailand; 3 Department of Anatomy, Faculty of Medicine, Khon Kaen University, Khon Kaen, 40002 Thailand; 4 Research Institute for Human High Performance and Health Promotion, Khon Kaen University, Khon Kaen, 40002 Thailand

**Keywords:** Molecular biology, Physiology

## Abstract

This study investigated the effects of nobiletin on cardiorenal changes and the underlying mechanisms involved in two-kidney, one-clip (2K-1C) hypertension. 2K-1C rats were treated with nobiletin (15 or 30 mg/kg/day) or losartan (10 mg/kg/day) for 4 weeks (*n* = 8/group). Nobiletin (30 mg/kg) reduced high levels of blood pressure and circulating angiotensin II and angiotensin-converting enzyme activity in 2K-1C rats. Left ventricular (LV) dysfunction and remodelling in 2K-1C rats were alleviated in the nobiletin-treated group (*P* < 0.05). Nobiletin reduced the upregulation of Ang II type I receptor (AT_1_R)/JAK (Janus kinase)/STAT (signal transducer and activator of transcription) protein expression in cardiac tissue of 2K-1C rats (*P* < 0.05). The reduction in kidney function, and accumulation of renal fibrosis in 2K-1C rats were alleviated by nobiletin (*P* < 0.05). Overexpression of AT_1_R and NADPH oxidase 4 (Nox4) protein in nonclipped kidney tissue was suppressed in the nobiletin-treated group (*P* < 0.05). The elevations in oxidative stress parameters and the reductions in antioxidant enzymes were attenuated in 2K-1C rats treated with nobiletin (*P* < 0.05). In summary, nobiletin had renin-angiotensin system inhibitory and antioxidant effects and attenuated LV dysfunction and remodelling via restoration of the AT_1_R/JAK/STAT pathway. Nobiletin also resolved renal damage that was related to modulation of the AT_1_R/Nox4 cascade in 2K-1C hypertension.

## Introduction

Renovascular hypertension is initiated by renal artery narrowing or renovascular stenosis and reduced renal blood flow. Goldblatt and coworkers first developed a renovascular hypertension model in dogs, and later, the two-kidney, one-clip (2K-1C) model was established in rats^1,2^. It is well recognized that the renin-angiotensin system (RAS) plays an important role in the development of hypertension in the 2K-1C model. Increases in circulating angiotensin II (Ang II) and angiotensin-converting enzyme (ACE) activity have been confirmed to occur in 2K-1C-induced hypertension in animals^3^. Subsequently, activation of RAS-mediated cardiovascular dysfunction and hypertrophy in renovascular hypertension has been noted^4^. Impairment of kidney function as well as kidney injury induced by stenosis of a renal artery have been reported in an animal model of renovascular hypertension. These are clearly associated with a high level of circulating Ang II^5,6^. Ang II, the key product of RAS, is a powerful vasoconstrictor that promotes an elevation in systemic vascular resistance and high blood pressure. The most pathological actions of Ang II on the heart, vessels and kidneys are mediated by local tissue Ang II type I receptor (AT_1_R)^7^. The decline in left ventricular function induced by 2K-1C modelling is associated with high levels of Ang II^8^. Several studies have reported overexpression of AT_1_R protein in cardiac tissue that is related to hypertrophy and remodelling in 2K-1C animals^9,10^. In kidney tissue, glomerular and tubulointerstitial damage in the nonclipped kidney associated with increased serum Ang II has been observed in 2K-1C rats^5^. Additionally, 2K-1C-induced hypertensive rats have kidney interstitial fibrosis and kidney dysfunction that are linked to overactivation of ROS production^11^.

One of the molecular mechanisms of RAS activation-induced organ damage has been proposed to be associated with oxidative stress since Ang II binding to AT_1_R can stimulate NADPH oxidase (Nox)^12^. It is well established that NADPH oxidase plays an important role in producing a reactive oxygen species, the superoxide anion radical (O_2_^·−^). 2K-1C rat models show oxidative stress, as evidenced by high levels of systemic malondialdehyde (MDA) and vascular O_2_^·−^ production as well as suppression of antioxidant enzymes^13,14^. A previous study has shown that hypertension-induced kidney injury in rats is mediated by activation of the AT_1_R/Nox4/oxidative stress signalling pathway^15^. Moreover, the molecular mechanism of Ang II-induced cardiac remodelling has been elucidated to be related to the activation of the AT_1_R/JAK (Janus kinase)/STAT (signal transducer and activator of transcription) signalling pathway^16^. The STATs are a family of transcription factors found in rat cardiac fibroblasts and myocytes^17^. Activation of the JAK/STAT3 pathway by Ang II has been confirmed to play an important role in hypertrophic growth of cardiac myocytes^18^. Recently, Ye et al. found that reducing the levels of phospho-STAT3 can attenuate Ang II-induced cardiac fibrosis and hypertrophy in cardiomyocytes^19^.

Nobiletin is a polymethoxylated flavone, a class of flavonoids mostly isolated from the peels of citrus species^20–22^. Substantial evidence has revealed a variety of pharmacological properties of nobiletin, such as anticancer, anti-insulin resistance, anti-inflammatory, and antioxidant activities^23^. Liu et al. demonstrated that nobiletin reduces the cell viability and proliferation of the breast cancer cell line MCF-7^24^. Nobiletin has nephroprotective effects by reducing oxidative stress in rats with acute kidney injury^25^. The antidiabetic effects of nobiletin have also been demonstrated: nobiletin restores blood glucose and insulin levels and ameliorates kidney dysfunction, tubular collagen deposition and injury in diabetic rats^26^. Additionally, the potential effects of nobiletin on the signs and complications of metabolic syndrome induced by a high-fat diet in rats have been revealed^27^. In rats with L-NAME-induced hypertension, nobiletin exhibits antihypertensive effects that are related to reduction of the sympathetic nerve-mediated contractile response, improvement of endothelial-dependent vascular relaxation, and alleviation of vascular remodelling via restoration of Nrf-2/HO-1 and MMP protein expression^28^. This study aimed to investigate the effects of nobiletin on cardiorenal structural and functional alterations and the mechanisms involved in 2K-1C rats.

## Results

### Effects of nobiletin or losartan on blood pressure and haemodynamic parameters

**Figure 1:**
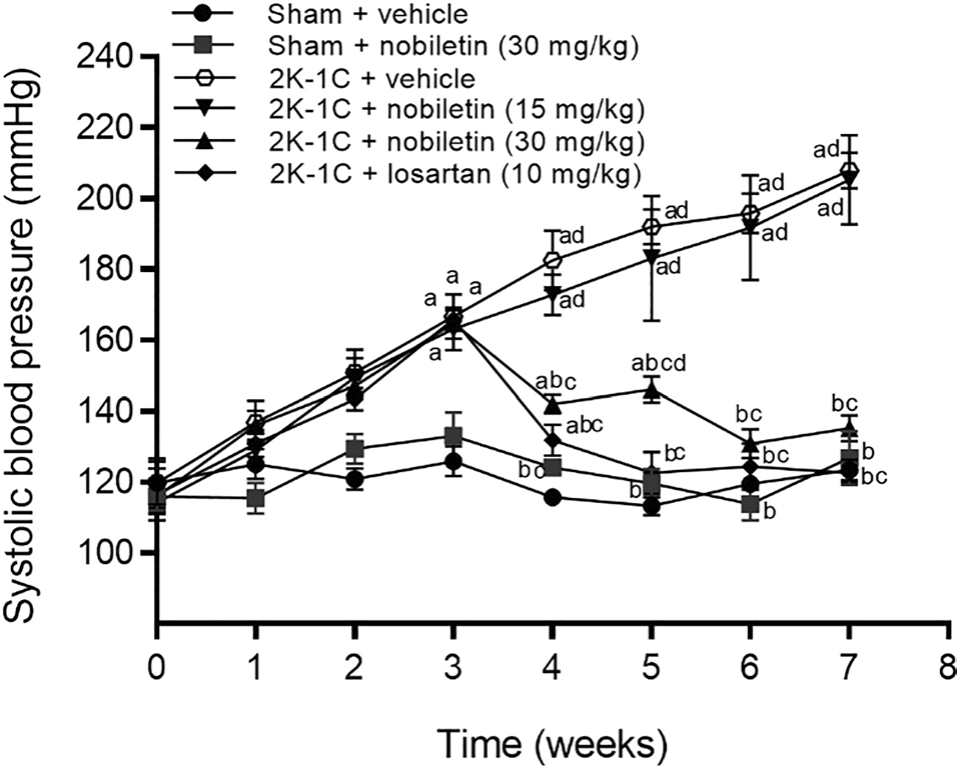
Effects of nobiletin or losartan on weekly systolic pressure (SP) in conscious 2K-1C rats. The data are expressed as the means ± SEMs. ^a^*P* < 0.05 vs. sham, ^b^*P* < 0.05 vs. 2K-1C, ^c^*P* < 0.05 vs. 2K-1C + nobiletin (15 mg/kg), ^d^*P* < 0.05 vs. sham + nobiletin (30 mg/kg). *2K-1C* two-kidney, one-clip.

Changes of systolic blood pressure (SP) throughout of 7 weeks of experiments are shown in Fig. 1. At the beginning of the experiment, SP was not different among groups. After 2K-1C and sham operation, SP progressively increased in the 2K-1C-operated groups and was significantly higher than that in the sham-operated group after 2 weeks of induction. Oral administration of nobiletin at a dose of 30 mg/kg for 4 weeks markedly decreased SP in hypertensive rats (*P* < 0.05), while nobiletin at a dose of 15 mg/kg had no effect on SP in hypertensive rats. In addition, nobiletin (30 mg/kg) did not show a hypotensive effect in sham-operated rats. Losartan (10 mg/kg), a positive control agent, significantly reduced SP in the hypertensive group (*P* < 0.05). Furthermore, the SP did not differ between the nobiletin (30 mg/kg) and losartan groups.

The haemodynamic parameters measured under anaesthesia in all experimental groups are shown in Table 1. SP, DP, MAP, PP and HR were higher in 2K-1C rats than in control rats (*P* < 0.05). Compared to the untreated 2K-1C group, the group treated with nobiletin at a dose of 30 mg/kg significantly exhibited significantly attenuated alterations in these parameters, except HR, (*P* < 0.05). In addition, losartan treatment attenuated 2K-1C-induced hypertension and haemodynamic alterations. However, HR was not significantly different among the groups of 2K-1C rats.

**Table 1:** Effects of nobiletin or losartan on haemodynamic parameters in 2K-1C rats under anaesthesia.

Parameter	Sham	2K-1C			
	Vehicle	Vehicle	Nobiletin 15 mg/kg	Nobiletin 30 mg/kg	Losartan 10 mg/kg
SP (mmHg)	122 ± 3.97	195.89 ± 9.50^a^	178.24 ± 10.94^a^	131.21 ± 2.09^b,c^	124.20 ± 2.53^b,c^
DP (mmHg)	79.89 ± 4.42	130.98 ± 6.39^a^	99.61 ± 3.82^b^	85.68 ± 2.87^b^	83.10 ± 2 .57^b^
MAP (mmHg)	93.93 ± 4.20	152.61 ± 7.32^a^	125.52 ± 4.75^a^^,^^b^	100.52 ± 2.34^b,c^	96.80 ± 2.35^b,c^
PP (mmHg)	42.11 ± 1.73	64.91 ± 4.07^a^	79.08 ± 11.03^a^	44.54 ± 2.57^b,c^	41.10 ± 2.12^b,c^
HR (beats/min)	361.89 ± 16.53	425.53 ± 16.7^a^	415.48 ± 23.65	364.62 ± 19.15	352.96 ± 7.56^b^

The data are expressed as the means ± SEMs.

*SP* systolic blood pressure, *DP* diastolic blood pressure, *MAP* mean arterial pressure, *PP* pulse pressure, *HR* heart rate, *2K-1C* two-kidney, one-clip.

^a^*P* < 0.05 vs. sham.

^b^*P* < 0.05 vs. 2K-1C.

^c^*P* < 0.05 vs. 2K-1C + nobiletin (15 mg/kg).

### Effects of nobiletin or losartan on organ weights

After 7 weeks of the experimental period, reduced final body weights of untreated 2K-1C rats compared to untreated sham rats were observed (*P* < 0.05; Table 2). Nobiletin at a dose of 30 mg/kg or losartan significantly improved the BW of 2K-1C rats. In addition, the HW/BW, VW/BW, and LVW/BW ratios were significantly increased in untreated 2K-1C rats compared with sham-operated rats (*P* < 0.05; Table 2). After treatment with nobiletin at a dose of 30 mg/kg or losartan, the elevations in these ratios were attenuated in 2K-1C rats (*P* < 0.05; Table 2). However, nobiletin at a dose of 15 mg/kg did not improve the BW or the ratios in 2K-1C rats.

**Table 2:** Effects of nobiletin or losartan on organ weight/body weight ratios in 2K-1C rats.

Parameter	Sham	2K-1C			
	Vehicle	Vehicle	Nobiletin 15 mg/kg	Nobiletin 30 mg/kg	Losartan 10 mg/kg
BW (g)	546.75 ± 8.58	433.71 ± 32.71^a^	503.00 ± 32.34	526.71 ± 10.29^b^	531.67 ± 16.44^b^
HW/BW (mg/g)	2.55 ± 0.05	3.79 ± 0.16^a^	3.71 ± 0.34^a^	2.69 ± 0.12^b,c^	2.71 ± 0.07^b,c^
VW/BW (mg/g)	2.17 ± 0.03	3.30 ± 0.14^a^	3.23 ± 0.42^a^	2.34 ± 0.12^b,c^	2.40 ± 0.09^b,c^
LVW/BW (mg/g)	1.66 ± 0.02	2.74 ± 0.12^a^	2.68 ± 0.226^a^	1.82 ± 0.11^b,c^	1.84 ± 0.09^b,c^
RKW/BW (mg/g)	3.37 ± 0.09	4.97 ± 0.20^a^	4.74 ± 0.27^a^	3.87 ± 0.17^b,c^	3.87 ± 0.14^b,c^
LKW/BW (mg/g)	3.35 ± 0.05	2.56 ± 0.14^a^	2.5 ± 0.37^a^	2.96 ± 0.24	3.02 ± 0.17

The data are expressed as the means ± SEMs.

*HW/BW* heart weight/body weight ratio, *VW/BW* ventricular weight/body weight ratio, *LVW/BW* left ventricular weight/body weight ratio, *RKW/BW* right kidney weight/body weight ratio, *LKW/BW* left kidney weight/body weight ratio, 2K-1C.

^a^*P* < 0.05 vs. sham.

^b^*P* < 0.05 vs. 2K-1C.

^c^ *P* < 0.05 vs. 2K-1C + nobiletin (15 mg/kg).

**Figure 2:**
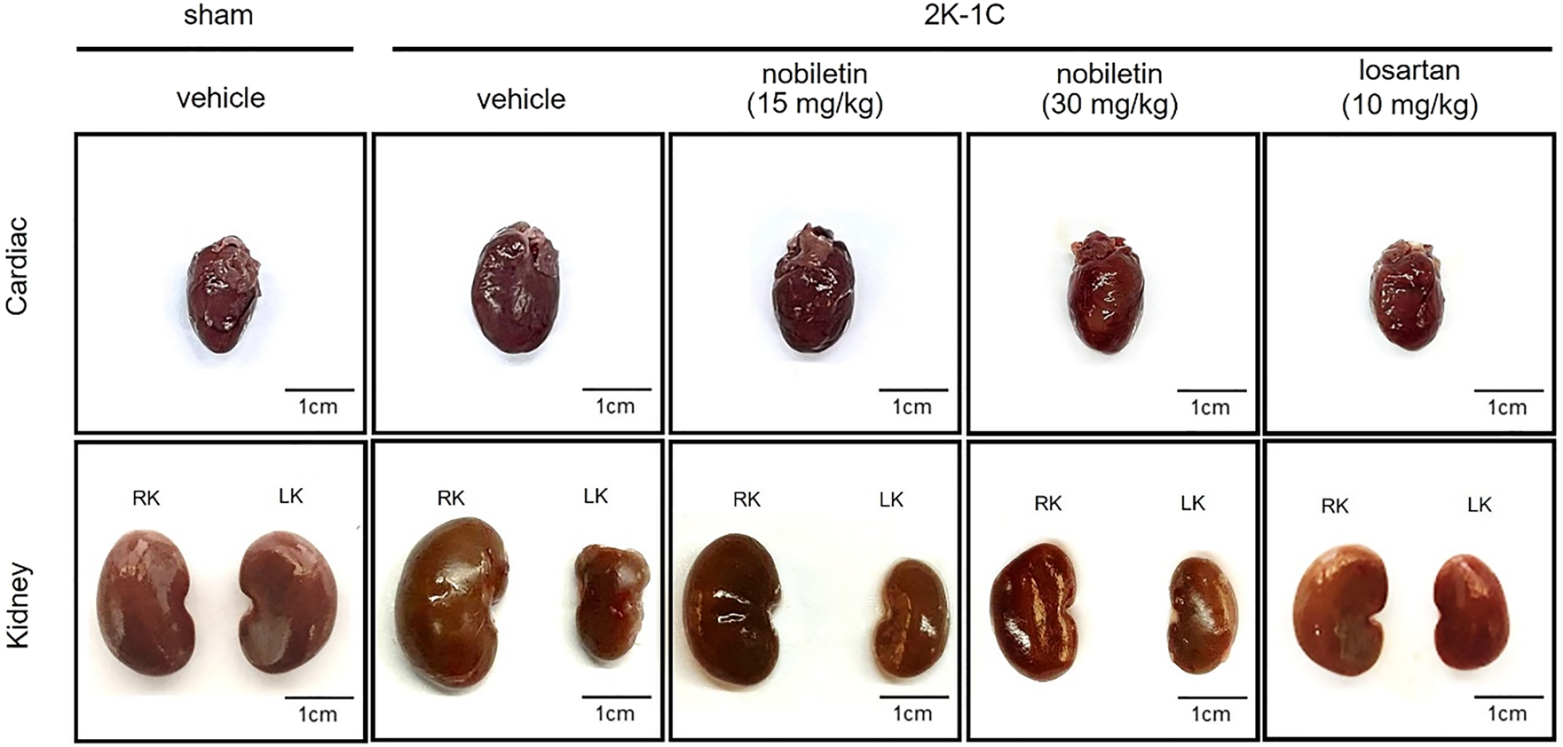
Representative images of the hearts (upper panel) and kidneys (lower panel) of 2K-1C rats after 7 weeks of the experiment (scale bar = 1 cm). *RK* right kidney (nonclipped side), *LK* left kidney (clipped side), *2K-1C* two-kidney, one-clip.

With regard to kidney weight, significant increases in RKW/BW and LKW/BW were found in 2K-1C rats compared to sham-operated rats (*P* < 0.05). Compared with no treatment, treatment with nobiletin at a dose of 30 mg/kg or losartan significantly reduced RKW/BW in 2K-1C rats (*P* < 0.05; Table 2). However, LKW/BW did not differ among the groups. Representative images of the hearts and kidneys are shown in Fig. 2.

### Effects of nobiletin and losartan on ACE activity and Ang II levels

**Figure 3:**
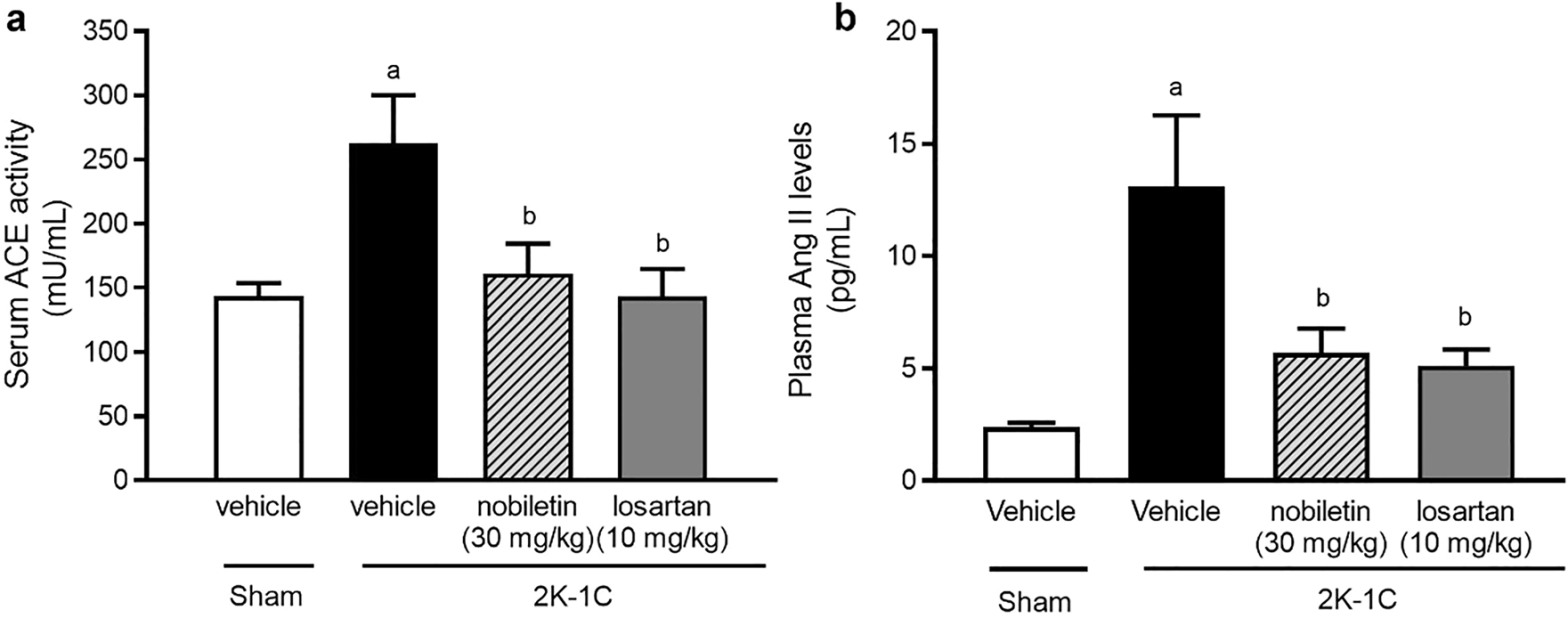
Effects of nobiletin or losartan on serum angiotensin-converting enzyme activity (ACE; (**a**)) and plasma angiotensin II levels (Ang II, (**b**)) in 2K-1C rats. The data are expressed as the means ± SEMs. ^a^*P* < 0.05 vs. sham, ^b^*P* < 0.05 vs. 2K-1C. *2K-1C* two-kidney, one-clip.

The levels of serum ACE activity and plasma Ang II were significantly increased in the untreated 2K-1C group compared with the sham group (*P* < 0.05). However, nobiletin or losartan treatment alleviated RAS overactivity, as supported by the significant reductions in serum ACE activity and plasma Ang II levels in 2K-1C rats treated with nobiletin at a dose of 30 mg/kg or losartan compared with the levels in sham rats (*P* < 0.05), as shown in Fig. 3.

### Effect of nobiletin or losartan on cardiac function

**Figure 4:**
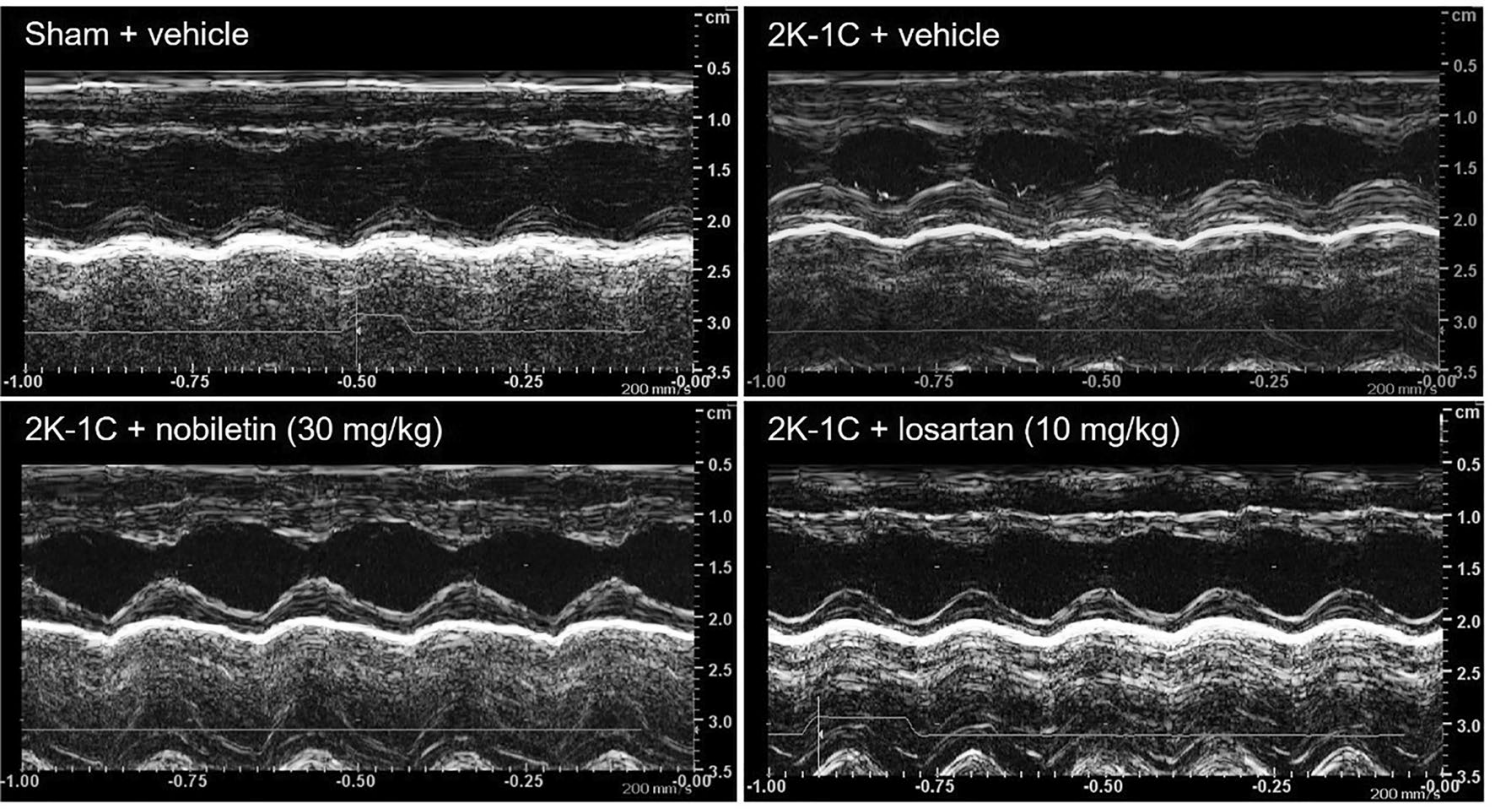
Representative tracings of transthoracic echocardiographs in 2K-1C rats. *2K-1C* two-kidney, one clip.

At 7 weeks after the 2K-1C operation, impairment of LV function was observed in the untreated 2K-1C group, represented by the elevations in IVSd, LVPWd, and ESV and reductions in %EF, SV, and %FS compared with the levels in the untreated sham group (*P* < 0.05; Table 3). Oral administration of nobiletin at a dose of 30 mg/kg or losartan for 4 weeks alleviated 2K-1C-induced LV dysfunction, supported by the recovery of ESV, EF, and FS compared with the levels in the untreated 2K-1C group (*P* < 0.05; Table 3). In addition, losartan treatment significantly reduced LVPWd compared with the value in the untreated 2K-1C group (*P* < 0.05; Table 3). Representative tracings of transthoracic echocardiographs in all groups of rats are shown in Fig. 4.

**Table 3:** Effects of nobiletin or losartan on cardiac function in 2K-1C rats.

Parameter	Sham	2K-1C		
	Vehicle	Vehicle	Nobiletin 30 mg/kg	Losartan 10 mg/kg
IVSd (mm)	1.61 ± 0.04	2.00 ± 0.08^a^	1.76 ± 0.09	1.64 ± 0.13^b^
IVSs (mm)	2.56 ± 0.05	2.85 ± 0.15	2.90 ± 0.19	2.63 ± 0.14
LVIDd (mm)	8.29 ± 0.02	8.20 ± 0.23	8.04 ± 0.13	8.29 ± 0.13
LVIDs (mm)	4.65 ± 0.07	5.33 ± 0.17^a^	4.39 ± 0.23^b^	4.65 ± 0.14^b^
LVPWd (mm)	1.73 ± 0.05	2.30 ± 0.02^a^	2.08 ± 0.11	1.64 ± 0.14^b^
LVPWs (mm)	2.66 ± 0.08	3.10 ± 0.23	2.98 ± 0.16	2.57 ± 0.25^b^
EDV (mL)	1.26 ± 0.08	1.16 ± 0.09	1.13 ± 0.05	1.23 ± 0.07
ESV (mL)	0.25 ± 0.02	0.36 ± 0.04^a^	0.22 ± 0.03^b^	0.25 ± 0.02^b^
EF (%)	79.73 ± 0.73	69.28 ± 0.97^a^	80.82 ± 1.97^b^	79.78 ± 0.81^b^
SV (mL)	1.00 ± 0.06	0.805 ± 0.05^a^	0.91 ± 0.03	0.99 ± 0.03^b^
FS (%)	43.75 ± 0.85	34.65 ± 0.58^a^	45.24 ± 2.35^b^	42.65 ± 0.68^b^

The data are expressed as the means ± SEMs.

*IVSd* interventricular septum thickness at diastole, *IVSs* interventricular septum thickness at systole, *LVIDd* left ventricular internal dimension at end-diastole, *LVIDs* left ventricular internal dimension at end-systole, *LVPWd* left ventricular posterior wall thickness at diastole, *LVPWs* left ventricular posterior wall thickness at systole, *EDV* end-diastolic volume, *ESV* end-systolic volume, *SV* stroke volume, *EF* ejection fraction, *FS* fractional shortening, *2K-1C* two-kidney, one-clip.

^a^*P* < 0.05 vs. sham.

^b^*P* < 0.05 vs. 2K-1C.

### Effect of nobiletin or losartan on collagen type I protein expression in cardiac tissue

**Figure 5:**
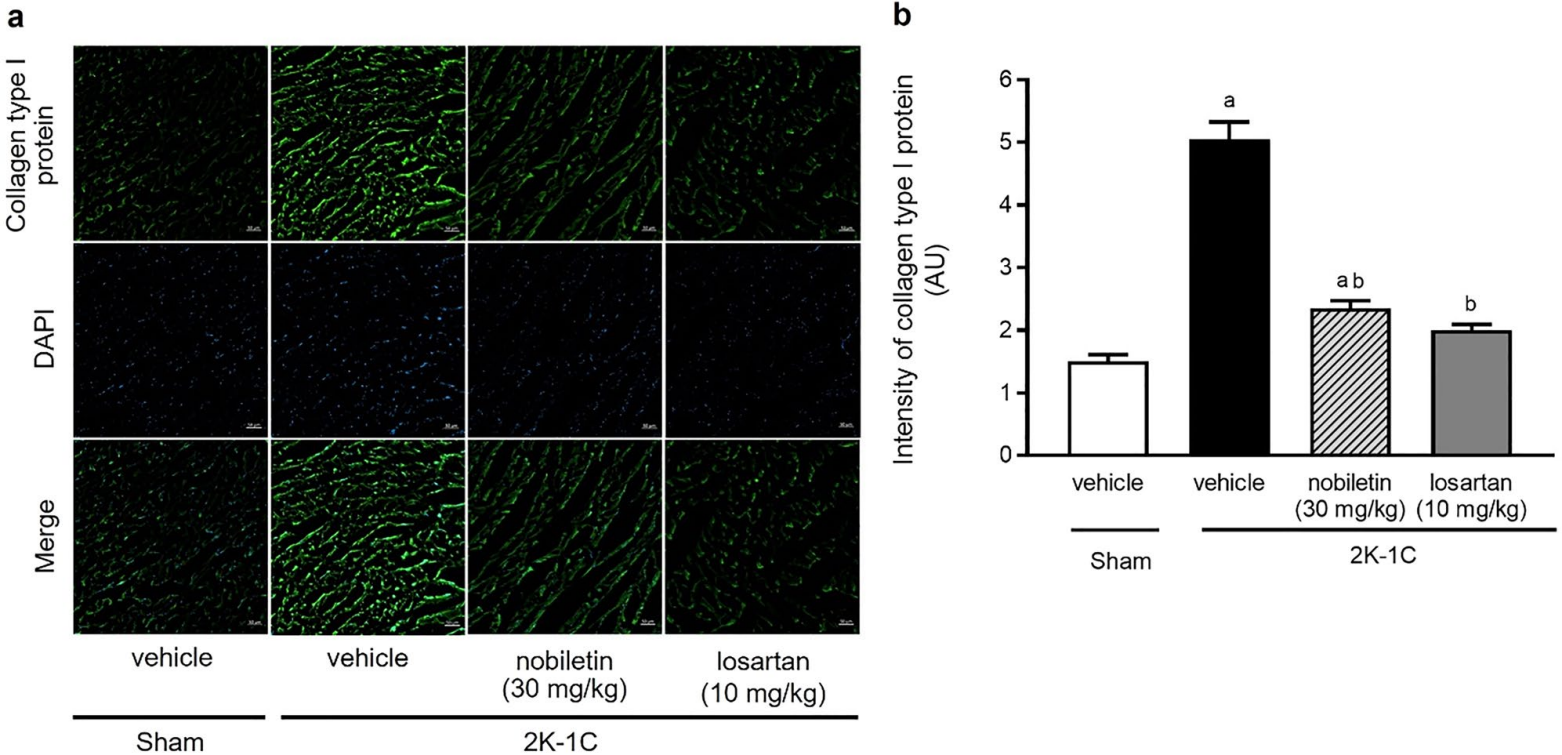
Representative images of the immunofluorescence of collagen type I (**a**) in left ventricular tissue (magnification is × 10, scale bar = 50 µm). Quantitative data for collagen type I (**b**) in cardiac tissue in 2K-1C rats. The data are expressed as the means ± SEMs. ^a^*P* < 0.05 vs. sham, ^b^*P* < 0.05 vs. 2K-1C. *2K-1C* two-kidney, one-clip.

The immunofluorescence images of collagen type I protein expression in the LV are shown in Fig. 5a. The fluorescence signals of collagen type I in the LV were significantly intensified in untreated 2K-1C rats compared with untreated sham rats (*P* < 0.05). However, treatment with nobiletin at a dose of 30 mg/kg or losartan for 4 weeks attenuated the overexpression of collagen type I in the LV observed in the untreated 2K-1C rats. These results were supported by the quantitative data for collagen type I protein, as shown in Fig. 5b. The intensity of collagen type I protein staining in the untreated 2K-1C group was significantly lower than that in the untreated sham group (*P* < 0.05).

### Effects of nobiletin and losartan on AT_1_R, JAK2, and STAT3 protein expression in cardiac tissue

**Figure 6:**
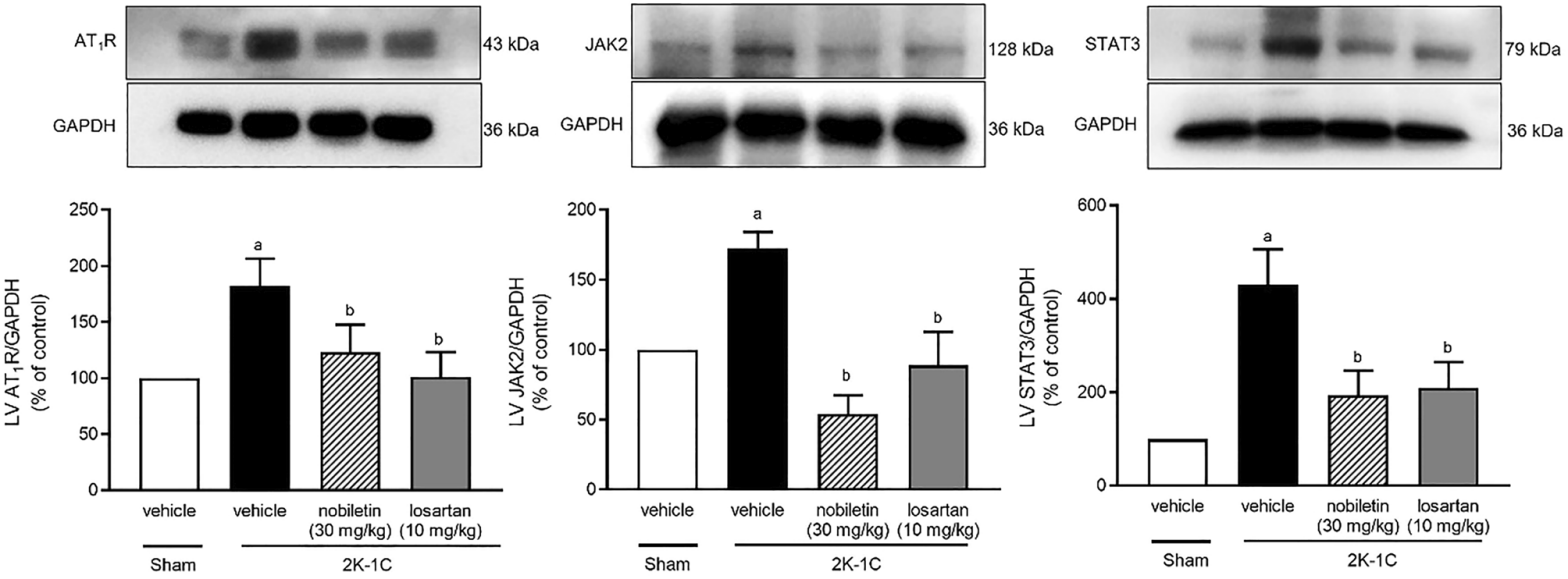
Effects of nobiletin or losartan on AT_1_R (n = 6) (**a**), JAK2 (n = 3) (**b**), and STAT3 (n = 6) (**c**) protein expression in cardiac tissue in 2K-1C rats. The data are expressed as the means ± SEMs. ^a^*P* < 0.05 vs. sham, ^b^*P* < 0.05 vs. 2K-1C. *2K-1C* two-kidney, one-clip. The blots were cut prior to hybridisation with antibodies during blotting, uncropped blots are included in Supplementary Information.

The protein expression levels of AT_1_R, JAK2, and STAT3 in cardiac tissue were higher in the untreated 2K-1C group than in the sham-operated group (*P* < 0.05). However, nobiletin or losartan significantly reduced the upregulation of AT_1_R, JAK2, and STAT3 expression in 2K-1C rats (*P* < 0.05), as shown in Fig. 6.

### Effects of nobiletin and losartan on kidney function

The 2K-1C operation induced kidney damage, as supported by the greater proteinuria and albuminuria in renovascular hypertensive rats than in control rats (*P* < 0.05, Table 4). In addition, the levels of serum creatinine were significantly higher, while urine creatinine and the eGFR were significantly lower, in 2K-1C rats than in sham-operated rats (*P* < 0.05; Table 4). The albumin/creatinine ratio was higher in hypertensive rats than in control rats. Oral administration of nobiletin or losartan ameliorated kidney damage by reducing albuminuria in 2K-1C rats compared with untreated 2K-1C rats (*P* < 0.05; Table 4). In addition, nobiletin and losartan significantly improved the eGFR and albumin/creatinine ratio in 2K-1C rats (*P* < 0.05; Table 4).

**Table 4:** Effects of nobiletin or losartan on kidney function in 2K-1C rats.

Parameter	Sham	2K-1C		
	Vehicle	Vehicle	Nobiletin 30 mg/kg	Losartan 10 mg/kg
Urine creatinine (mg/dL)	137.42 ± 15.82	67.02 ± 17.26^a^	116.68 ± 15.67	116.88 ± 12.81
Urine flow (mL/min)	0.02 ± 0.003	0.015 ± 0.002	0.01 ± 0.001	0.02 ± 0.004
Serum creatinine (mg/dL)	0.97 ± 0.11	1.38 ± 0.09^a^	0.81 ± 0.48^b^	0.81 ± 0.08^b^
eGFR (mL/min)	2.95 ± 0.32	0.91 ± 0.35^a^	1.82 ± 0.34^b^	2.47 ± 0.33^b^
Albuminuria (mg/dL)	18.81 ± 3.5	187.59 ± 11.82^a^	117.61 ± 2.08^a,b^	86.06 ± 9.33^a,b^
Albumin/creatinine ratio	0.14 ± 0.03	4.58 ± 1.48^a^	1.26 ± 0.17^b^	0.88 ± 0.09^b^

The data are expressed as the means ± SEMs.

*2K-1C* two-kidney, one-clip, *eGFR* estimated glomerular filtration rate.

^a^*P* < 0.05 vs. sham.

^b^*P* < 0.05 vs. 2K-1C.

### Effects of nobiletin and losartan on kidney morphology

**Figure 7:**
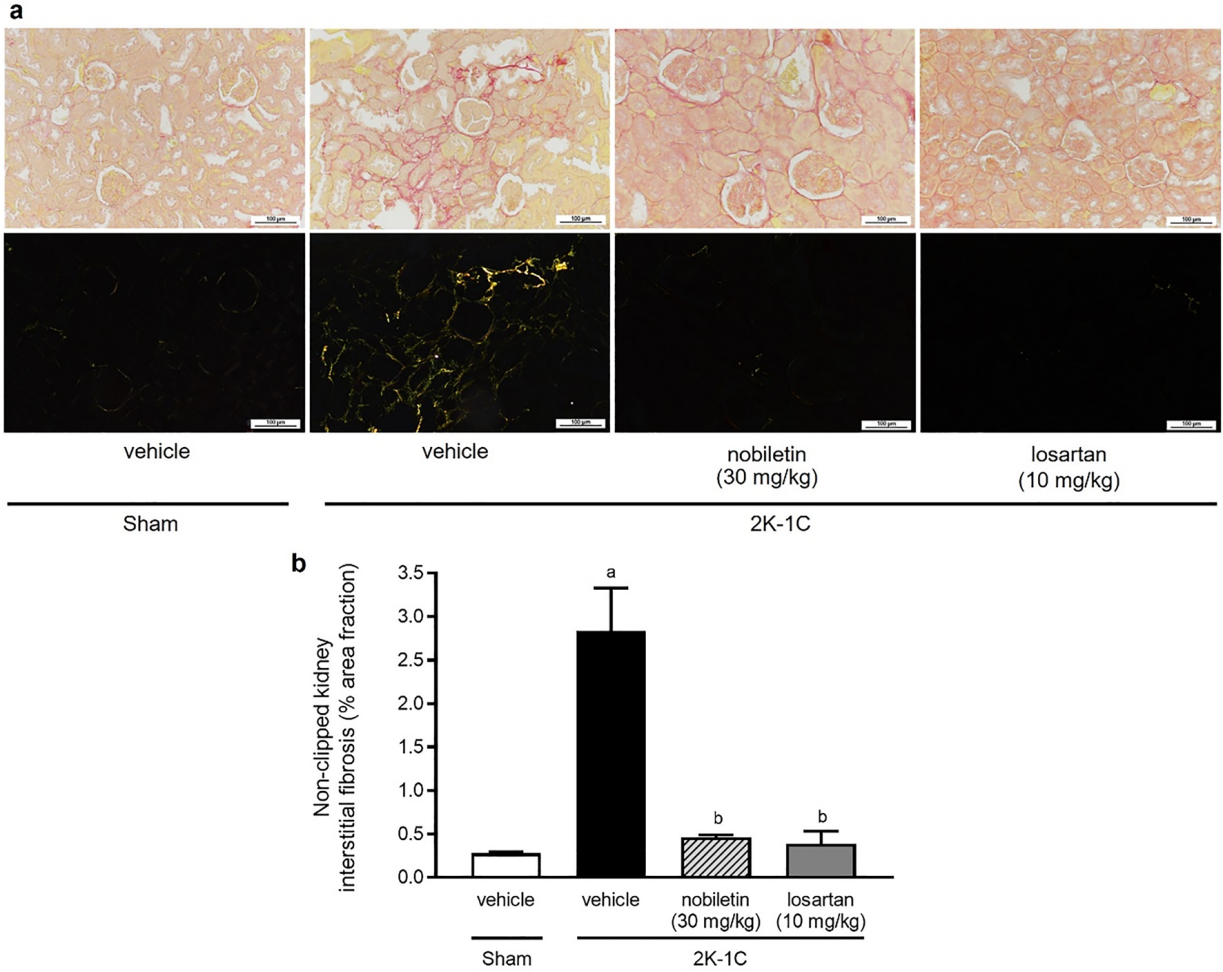
Effects of nobiletin or losartan on renal fibrosis in 2K-1C rats. Representative images of nonclipped kidney sections (**a**) stained with picrosirius red. All pictures were imaged under a light microscope (upper panel) and a polarized light microscope (lower panel) (magnification is × 20, scale bar = 100 µm). Quantitative data for the level of fibrosis (**b**) in nonclipped kidney tissue in 2K-1C rats. The data are expressed as the means ± SEMs. ^a^*P* < 0.05 vs. sham, ^b^*P* < 0.05 vs. 2K-1C, *2K-1C* two-kidney, one-clip.

The interstitial fibrosis accumulation of nonclipped kidneys in the untreated 2K-1C group was more intense than that in the other groups (Fig. 7a), accompanied by an increase in the % area fraction of interstitial fibrosis (*P* < 0.05; Fig. 7b). However, nobiletin or losartan treatment ameliorated the fibrotic changes of the kidney observed in the untreated 2K-1C group (*P* < 0.05).

### Effects of nobiletin and losartan on kidney AT_1_R and Nox4 protein expression

**Figure 8:**
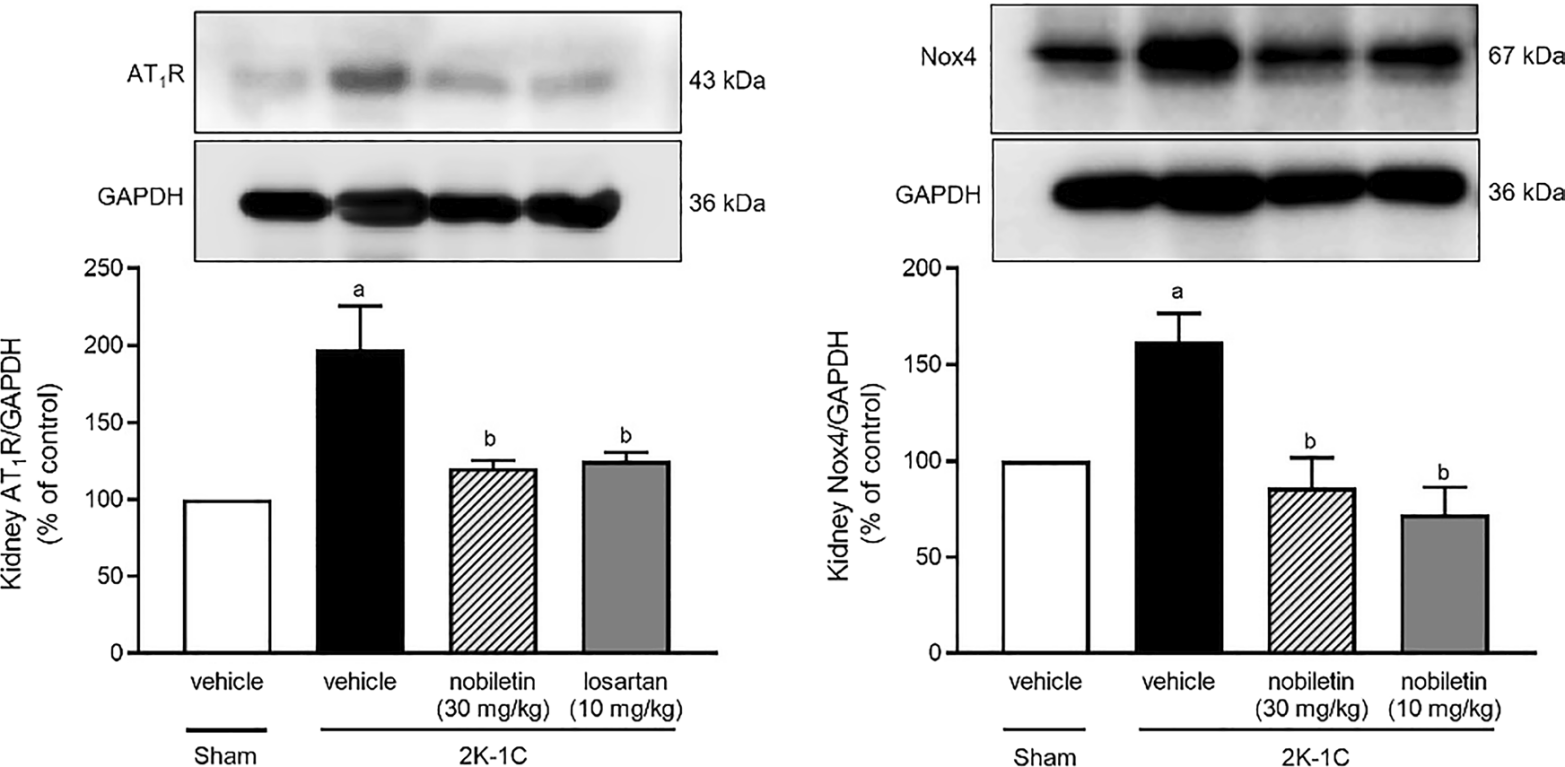
Effects of nobiletin or losartan on AT_1_R (n = 4) (**a**) and Nox4 (n = 5) (**b**) protein expression in nonclipped kidney tissue in 2K-1C rats. The data are expressed as the means ± SEMs. ^a^*P* < 0.05 vs. sham, ^b^*P* < 0.05 vs. 2K-1C. *2K-1C* two-kidney, one-clip. The blots were cut prior to hybridisation with antibodies during blotting, uncropped blots are included in Supplementary Information.

Significant increases in AT_1_R and Nox4 protein expression in kidney tissue were observed in the untreated 2K-1C group compared with the untreated sham group (*P* < 0.05). However, nobiletin at a dose of 30 mg/kg or losartan significantly reduced the kidney protein expression of AT_1_R and Nox4 compared with that in untreated 2K-1C rats (*P* < 0.05), as shown in Fig. 8.

### Effects of nobiletin and losartan on oxidative stress markers and endogenous antioxidant enzymes

**Figure 9:**
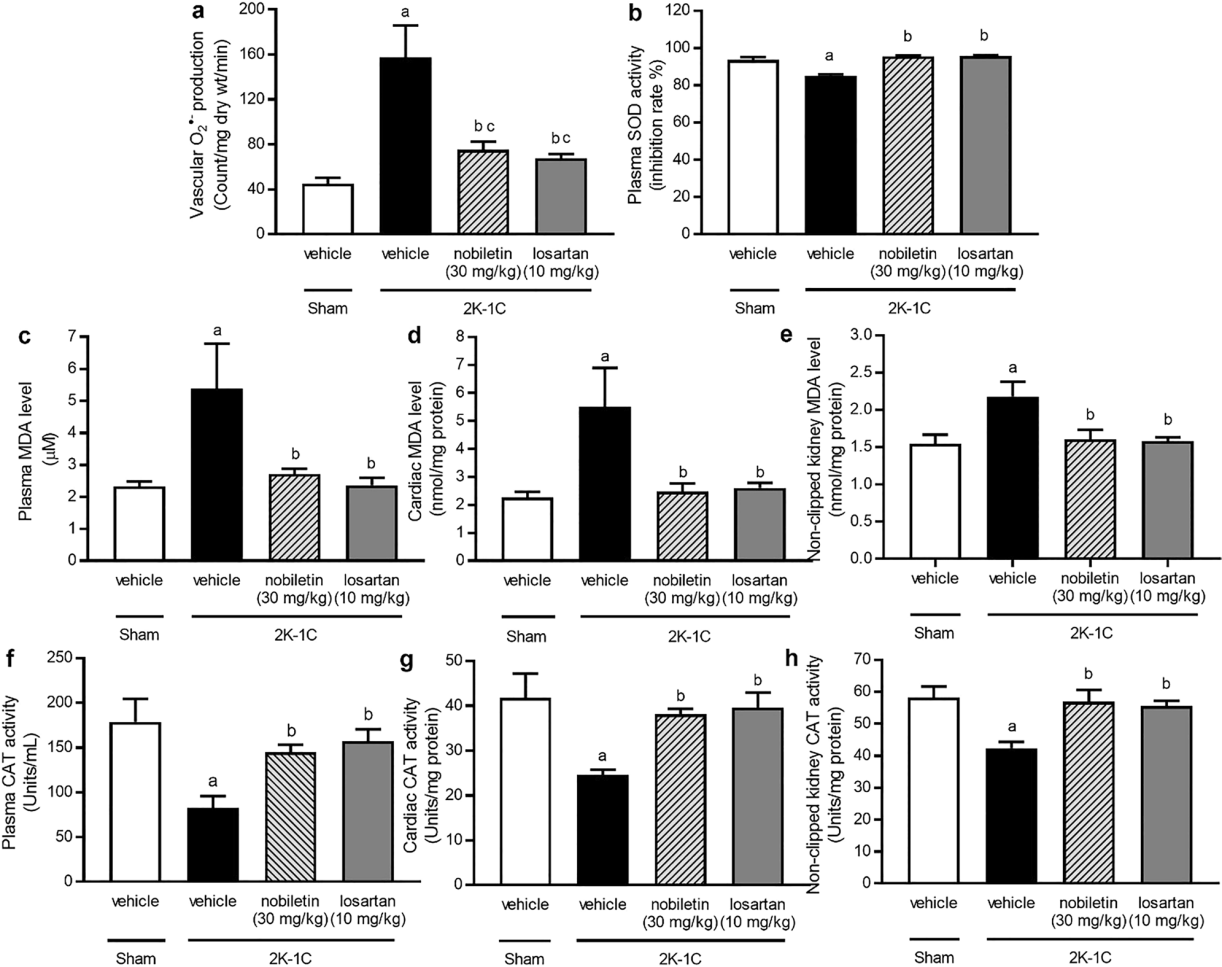
Effects of nobiletin or losartan on vascular O_2_^·−^ production (**a**), plasma superoxide dismutase (SOD) activity (**b**), plasma malondialdehyde (MDA) levels (**c**), cardiac MDA levels (**d**), nonclipped kidney MDA levels (**e**), plasma catalase (CAT) activity (**f**), cardiac CAT activity (**g**), and nonclipped kidney CAT activity (**h**) in 2K-1C rats. The data are expressed as the means ± SEMs. ^a^*P* < 0.05 vs. sham, ^b^*P* < 0.05 vs. 2K-1C. *2K-1C* two-kidney, one-clip.

At 7 weeks after the 2K-1C operation, elevated vascular O2^·−^ production and increased MDA levels in plasma, cardiac tissue, and nonclipped kidney tissue were observed in the untreated 2K-1C group compared with the sham-operated group (*P* < 0.05). In addition, significant reductions in plasma CAT and SOD, cardiac CAT, and nonclipped kidney CAT activity were observed in the untreated 2K-1C group compared with the untreated sham group (*P* < 0.05). Nobiletin and losartan treatments, however, improved oxidative status by reducing oxidative stress marker levels and increasing endogenous antioxidant enzyme activity in 2K-1C rats (*P* < 0.05), as shown in Fig. 9.

## Discussion

We found that nobiletin alleviated haemodynamic changes and normalized heart and kidney weights in 2K-1C hypertensive rats. High levels of serum ACE activity and plasma Ang II were reduced in 2K-1C rats treated with nobiletin. Impairment of cardiac function, as indicated by reductions in %EF and %FS, was observed in the hypertensive group, but this impairment was ameliorated by nobiletin treatment. Nobiletin also mitigated LV hypertrophy and fibrosis in 2K-1C hypertensive rats. These effects were relevant to suppression of the upregulation of AT_1_R/JAK2/STAT3 protein expression in cardiac tissue after treatment with nobiletin. The induction of kidney injury by left renal artery occlusion was supported by a reduction in the eGFR, an increase in albuminuria and accumulation of interstitial fibrosis in nonclipped kidney tissue. This kidney dysfunction and fibrosis in 2K-1C rats were ameliorated after nobiletin treatment. The protein expression of AT_1_R/Nox4 was upregulated in nonclipped kidney tissue, while in 2K-1C rats treated with nobiletin did not find the upregulation of these protein. The antioxidant effects of nobiletin were observed in 2K-1C rats since it reduced systemic and organ oxidative stress by reducing plasma and tissue MDA and O_2_^·−^ generation as well as recovering CAT and SOD activities. Losartan was a positive control in this experiment, and it exerted biological activity similar to that of nobiletin.

Nobiletin can attenuate haemodynamic alterations in renovascular hypertensive rats. The results demonstrated that nobiletin at a dose of 30 mg/kg was an effective dose. The 2K-1C procedure establishes what is known as RAS activation-induced high blood pressure. Several studies have reported activation of the RAS after unilateral occlusion of the renal artery in rats and mice^29–31^. The antihypertensive effect of nobiletin in the present study was related to a reduction in ACE activity and subsequent reductions in circulating Ang II levels. This study might be the first to report the inhibitory effects of nobiletin on ACE activity in 2K-1C hypertensive rats. The findings are consistent with many studies showing that other citrus flavonoids, such as hesperidin, diosmetin and naringenin, inhibit ACE activity^32–34^. Nobiletin also showed antioxidant effects in this animal model since it reduced systemic lipid peroxidation and restored endogenous antioxidant enzyme activity levels. These results are supported by several studies showing that nobiletin exhibits antioxidant properties both in vitro and in vivo^28,35^. There is strong evidence to confirm that oxidative stress is required to sustain high blood pressure in the rat model of 2K-1C^36^. Therefore, the antioxidant properties of nobiletin might have partially mediated the antihypertensive effect of nobiletin in 2K-1C rats in the present study. After 7 weeks of chronic RAS activation, the BW in 2K-1C rats was reduced comparing to sham group. The effect of a 2K-1C procedure on BW was controversy. It was not different from control rats^11,37^ while some studies showed reduction of BW in 2K-1C rats^38–40^. The reduction of BW in 2K-1C rats in the present study was consistent with previous study^38–40^. It is well known that BW is influenced by food intake and energy expenditure. We observed that food intake was not different among group. It is possible that increased energy expenditure might cause BW loss in 2K-1C rats in the present study. This was supported by the study that Ang II promotes the sympathetic outflow to increase energy expenditure and contribute to weigh loss in rats^41^. Nobiletin normalized rat BW might be related with suppression of RAS activation in 2K-1C rats.

Impairment of LV function and reductions in %EF and %FS were present in rats with high blood pressure induced by the 2K-1C operation in the present study. This LV dysfunction might have been caused by LV morphological changes that were indicated by LV remodelling in the hypertensive rats. LV hypertrophy was indicated by the elevations in HW/BW, VW/BW, LVW/BW, IVSd, and LVPWd, whereas LV fibrosis was also indicated by the increased intensity of LV collagen type I protein expression. However, nobiletin treatment alleviated LV abnormalities in 2K-1C hypertensive rats. This finding is consistent with a previous review showing that LV hypertrophy and fibrosis are commonly found in pathologic hearts, contributing to LV dysfunction^42,43^. Cardiac hypertrophy is mainly caused by an adaptive response to chronic high-pressure load to preserve cardiac output^44^. Additionally, the nonhemodynamic factor Ang II might participate in the development of LV remodelling^7^. Therefore, nobiletin may have improved cardiac function in this study through mechanisms related to its antihypertensive and ACE-inhibitory effects. The consequence of the reduction in RAS activation affected the Ang II/AT_1_R/JAK2/STAT3 signalling pathway. This study found that overexpression of AT_1_R/JAK2/STAT3 in 2K-1C rats mediates the cardiac remodelling process. There is evidence to show the association between AT_1_R/STAT3 and cardiac hypertrophy progression^45^. Additionally, many studies have provided evidence that Ang II is a growth factor that mediates the cardiac remodelling process via activation of AT_1_R, JAK2 and STAT3 in cardiac tissue^17,46,47^. It is possible that the molecular mechanisms of the beneficial effects of nobiletin on LV remodelling and function in the present study were associated with suppression of the AT_1_R/JAK2/STAT3 signalling pathway in 2K-1C rats.

It is well documented that 2K-1C rats have kidney injury, as indicated by accumulation of kidney interstitial fibrosis in nonclipped kidneys and increases in albuminuria and the albumin-to-creatinine ratio. An elevation in KW/BW on the nonclipped side was observed in 2K-1C rats, suggesting kidney hypertrophy. In addition, kidney function was impaired in 2K-1C rats, as indicated by the high levels of serum creatinine and the low eGFR values in these rats. The induction of kidney injury by renal artery occlusion has been supported by numerous studies^48,49^. Lee et al. reported that 2K-1C-induced ACE/Ang II/AT_1_R overactivation causes kidney interstitial fibrosis and impairs kidney function, as indicated by high concentrations of serum creatinine, albuminuria, and albumin/creatinine ratio levels^11,50^. Our results showed that nobiletin alleviated 2K-1C-induced kidney abnormalities. This effect was associated with suppression of RAS activation and kidney oxidative stress. Furthermore, the finding suggested that oxidative stress in kidney tissue might have been the consequence of Ang II action on its receptor, AT_1_R. We found upregulation of AT_1_R and Nox4 protein expression in nonclipped kidneys. There is increasing evidence to support the idea that Nox subunit 4, or Nox4, is located in kidney tissue and contributes to oxidative stress and the pathology of kidney injury^15,51,52^. We found that 2K-1C rats that received nobiletin had recovered AT_1_R and Nox4 protein expression in nonclipped kidneys. Nobiletin has been reported to suppress Nox2 or gp91phox to alleviate cardiac hypertrophy^53^.

Losartan is an angiotensin II receptor blocker that is widely recommended for hypertension treatment^54^. It was used as a positive control agent in this study. It was found that losartan reduced blood pressure and attenuated cardiorenal alterations in 2K-1C rats. Losartan also had inhibitory effects on RAS activation and oxidative stress in 2K-1C rats. Angiotensin II receptor blockers can prevent and alleviate organ damage^55,56^. The mechanisms of action of losartan on cardiorenal tissue in this study were related to blockade of RAS and subsequent suppression of the AT_1_R/JAK/STAT signalling pathway as well as the AT_1_R/Nox4 cascade. It has been reported that losartan also acts as an antioxidant and anti-inflammatory agent^57^. In an animal model of NO depletion-induced hypertension, losartan reduced blood pressure and cardiorenal damage by reducing oxidative stress^58^.

In summary, nobiletin showed beneficial effects on cardiorenal abnormalities induced by the 2K-1C procedure, similar to the losartan used in the experiment. It ameliorated LV dysfunction and remodelling by reducing oxidative stress as well as by suppressing the Ang II/AT_1_R/JAK/STAT signalling pathway in cardiac tissue. Furthermore, the RAS-inhibitory and antioxidant properties of nobiletin resolved renal injury in a manner related to the AT_1_R/Nox4 pathway in renovascular hypertensive rats.

## Materials and methodology

### Drugs and animals

Nobiletin was obtained from INDOFINE Chemical Company, Inc. (NJ, USA). Losartan (Cozaar 50 mg) was obtained from MSD. Merck & Co., Inc., (NJ, USA).

This study used male Sprague–Dawley rats (5–6 weeks of age weighing 160–180 g) that were provided by Nomura Siam International Co., Ltd., Bangkok, Thailand. The rats were raised in an HVAC (heating, ventilation and air-conditioning)-equipped room (23 ± 2 °C) with a 12 h dark–light cycle at the Northeast Laboratory Animal Center. All animal procedures complied with the standards for the care and use of experimental animals and were approved by the Animal Ethics Committee of Khon Kaen University, Khon Kaen, Thailand (IACUC-KKU-73/63). This study is reported in accordance with Animal Research: Reporting of In Vivo Experiments (ARRIVE) guidelines.

### Induction of 2K-1C hypertension and experimental protocols

After a week of acclimatization, hypertension was induced in the rats by reducing renal blood flow. The 2K-1C procedure was performed under anaesthesia with intraperitoneal injection of xylazine (5 mg/kg) followed by zoletil (25 mg/kg). A silver clip (0.2 mm i.d.) was carefully verified and placed on the left renal artery. Sham-operated rats were also used. Three weeks after the operation, the rats that had a systolic blood pressure (SP) > 160 mmHg were included as 2K-1C hypertensive rats^29^. There were six experimental groups (*n* = 8/each group) as follows:

1. One sham group was given vehicle (propylene glycol, 1.5 mL/kg).
2. Another sham group was given 30 mg/kg nobiletin.
3. A 2K-1C group was given vehicle (propylene glycol, 1.5 mL/kg).
4. A 2K-1C group was given 15 mg/kg nobiletin.
5. A 2K-1C group was given 30 mg/kg nobiletin.
6. A 2K-1C group was given losartan (10 mg/kg).

Nobiletin, losartan and propylene glycol were intragastrically administered daily by using a gavage feeding tube for 4 weeks during the experimental period. Rats that treated nobiletin and losartan received propylene glycol, 1.5 mL/kg.

### Assessment of systolic blood pressure during 7 weeks of experiments

SP was detected once a week without anaesthesia using noninvasive tail cuff plethysmography (IITC/Life Science Instrument Model 229 and Model 179 amplifier: Woodland Hills, CA, USA). Conscious rats were placed on a restrainer and allowed to acclimate prior to blood pressure measurement. The measurement was repeated 3 times, and the results are expressed as the mean values from each rat.

### Cardiac function and intraarterial blood pressure measurement

On the last day of experiment, all rats were anaesthetized with thiopental sodium (60 mg/kg, i.p.). Their chests were shaved and cleaned and they were placed on one side on a specially designed apparatus. LV function was assessed by echocardiogram using a GE Logiq S7 Ultrasound Machine (GE Healthcare, WI, USA). anaesthetized The LV function and structure were measured from the two-dimensional short-axis view, and M-mode tracings were recorded for the LV internal dimension at end-diastole (LVIDd), LV internal dimension at end-systole (LVIDs), interventricular septum thickness at diastole (IVSd), interventricular septum thickness at systole (IVSs), LV posterior wall thickness at diastole (LVPWd), LV posterior wall thickness at systole (LVPWs), end diastolic volume (EDV), end systolic volume (ESV), and stroke volume (SV) from three consecutive cardiac cycles. The LV fractional shortening (% FS) was calculated with the following equation: % LVFS = [(LVIDd − LVIDs)/LVIDd] × 100.

After echocardiogram, their femoral arteries were cannulated using polyethylene tubes. Baseline values of SP, diastolic blood pressure (DP), mean arterial pressure (MAP), and heart rate (HR) were continuously monitored for 30 min through a pressure transducer and recorded using Acknowledge Data Acquisition software (Biopac Systems Inc., Santa Barbara, CA, USA). After hemodynamic assessment, the animals were sacrificed by exsanguinations, collecting blood samples from abdominal aorta. The blood samples were collected in ethylenediaminetetraacetic acid tubes to prepare plasma and normal test tube to prepare serum. Tissue samples including heart and kidney were collected by dissection. All tissues were washed with saline solution and weighed. Data were expressed as a relation between regional tissue weights (g)/100 g BW.

### Angiotensin-converting enzyme activity and angiotensin II measurements

Serum ACE activity was evaluated using a fluorescence assay, as previously described, with some modifications^3^. The serum of each rat was mixed with hippuryl-l-histidyl-l-leucine (HHL) in assay buffer and then incubated at 37 °C for 30 min. After that, NaOH was added to stop the reaction, and the product of the reaction was fluorogenically labelled with O-phthaldialdehyde (OPA). The fluorescence was read at an excitation wavelength of 355 nm and an emission wavelength of 535 nm using a fluorescent plate reader.

Ang II levels were detected in plasma following the standard procedure recommendations of an Angiotensin II EIA Kit (RAB0010; Sigma–Aldrich, Merck KGaA, Darmstadt, Germany).

### Kidney function and albuminuria measurements

All rats were housed in metabolic cages for 24 h (Pestel et al.) for blood and urine collection. After urine collection, blood samples were obtained from the lateral tail veins. Serum and urine creatinine levels were evaluated using a creatinine assay kit (ab65340; Abcam Plc, Cambridge, U.K.). The estimated glomerular filtration rate (eGFR) was obtained from creatinine clearance measurement in ml/min as follows: eGFR = (U_cr_ × V)/P_cr_, U_cr_ = creatinine concentration in urine (mg/dL), V = urine flow rate (mL/min), and P_cr_ = creatinine concentration in plasma or serum (mg/dL)^59^.

The albuminuria levels were evaluated using a rat albumin ELISA Kit (ab108789; Abcam Plc, Cambridge, U.K.). The albuminuria results are expressed in ng/mL. Thereafter, urine creatinine and urine albumin levels were calculated as the urinary albumin-to-creatinine ratio to indicate the level of kidney damage.

### Picrosirius red staining in kidney tissue

To investigate the level of renal interstitial fibrosis, nonclipped kidneys were fixed with 4% paraformaldehyde for 24 h. Thereafter, the renal tissues were routinely processed, embedded in paraffin, and sectioned at 2 µm. Renal interstitial fibrosis was evaluated by picrosirius red staining. Images were captured using an Eclipse Ci-POL polarised light microscope (Nikon Tokyo, Japan). The level of kidney interstitial fibrosis was analysed using ImageJ morphometric software (NIH), and the result is expressed as a percentage of the area fraction^60^.

### Immunofluorescence in cardiac tissue

Heart tissues were fixed in 4% paraformaldehyde for 48 h at 4 °C. Thereafter, the tissues were transferred to 30% sucrose in phosphate-buffered saline (PBS) at 4 °C overnight. The heart tissues were embedded in embedding medium for frozen tissue specimens (4583, Sakura Finetek USA, Inc., Torrance, CA, USA) and cut into 10 µm thick sections at − 25 °C by using a Cryostat Microm HM 525 instrument (Thermo Fisher Scientific, Walldorf, Germany).

After that, the tissue sections were processed as previously described^61^. Briefly, the slides were processed and incubated with the primary antibody anti-collagen type I (ab34710, Abcam, Plc, Cambridge, U.K., dilution 1:200) at 4 °C overnight. After primary antibody incubation, Alexa Fluor^®^ 488 (ab150077, Abcam, Plc, Cambridge, U.K., dilution 1:1000) was used as the secondary antibody in a 2 h incubation at 37 °C, and the nuclei were stained using DAPI (D9542, Sigma–Aldrich, USA, dilution 1:1000) for 10 min at 37 °C. Thereafter, the slides were mounted, and fluorescence was visualized with a laser scanning confocal microscope (Carl Zeiss, Germany) with an appropriate filter. Confocal imaging was detected by a 10× objective lens and acquired with 1024 × 1024 pixels. The fluorescence intensity values of collagen type I and DAPI (average fluorescence intensity per pixel) were analysed by Zeiss Zen Blue analysis wizard software.

### Western blot analysis of AT_1_R, Nox4, JAK2, and STAT3 protein expression

The levels of AT_1_R and Nox4 protein expression in nonclipped kidney tissue and AT_1_R, JAK2, and STAT3 protein expression in cardiac tissue were evaluated by using the Western blot method. The tissue samples were electrophoresed through a sodium dodecyl sulfate polyacrylamide gel and transferred onto a polyvinylidenedifluoride (PVDF) membrane. Membranes were cut prior to hybridisation with antibodies during blotting. A mouse monoclonal antibody against AT_1_R (G-3) (SC-515884, dilution 1:1000), mouse monoclonal IgG_2b_ antibody against JAK2 (C-10) (SC-390539, dilution 1:1000) (Santa Cruz Biotechnology, Inc., Santa Cruz, CA, USA), mouse monoclonal IgG_2a_ antibody against STAT3 (124H6, dilution 1:800) (Cell Signaling Technology, Inc., Danvers, USA), rabbit monoclonal antibody against NADPH oxidase 4 (ab133303, dilution 1:1000), and mouse monoclonal antibody against GAPDH (ab133303, dilution 1:4000) (Abcam, Plc, Cambridge, UK) were used. GAPDH was used as a protein loading control to compare the intensities of protein expression. Densitometric analysis was performed using an ImageQuantTM 4000 imager (GE Healthcare Life Science, Piscataway, NJ, USA). The data are expressed as a percentage of the target protein (actin) expression level compared to that in the control groups.

### Measurements of oxidative stress markers

O_2_^·−^ production was detected in carotid arteries using lucigenin-enhanced chemiluminescence based on a technique described previously^62^. MDA production in plasma, nonclipped kidney tissue, and LV tissue was measured with a thiobarbituric acid-reactive substances (TBARS) assay using a spectrophotometric method as previously described^63^.

For detection of endogenous antioxidant enzymes and CAT enzyme activity in plasma, nonclipped kidney tissue, and LV tissue, the protocol followed previous reports^64,65^. In addition, plasma SOD activity was measured by colorimetric analysis using a spectrophotometer with corresponding detection kits (Sigma–Aldrich, Merck KGaA, Darmstadt, Germany) according to the manufacturer’s protocols.

### Statistical analysis

The results are shown as the mean ± the standard error of the mean (SEM). One-way analysis of variance (ANOVA) followed by Tukey’s test was used to analyse the differences among groups. The data were considered to show a statistically significant difference when the p value was less than 0.05.

## Supplementary Information

Supplementary Information.

## Data Availability

The datasets used and/or analysed during the current study available from the corresponding author on reasonable request.
