# Supplementary material for: Nobiletin resolves left ventricular and renal changes in 2K-1C hypertensive rats
Source: Sci Rep. 2022 Jun 3;12:9289. doi: 10.1038/s41598-022-13513-6 (PMC9166784; doi:10.1038/s41598-022-13513-6)
Supplement: Supplementary file 1 — Supplementary Information. [file 41598_2022_13513_MOESM1_ESM.docx]

Nobiletin Resolves Left Ventricular and Renal Changes in 2K-1C Hypertensive Rats

Metee Iampanichaku^1^, Anuson Poasakate^1^, Prapassorn Potue^1^, Siwayu Rattanakanokchai^2^, Putcharawipa Maneesai^1,4^, Parichat Prachaney^3,^ Wannapa Settheetham-Ishida^1^, Poungrat Pakdeechote^1,4,^*

^1^Department of Physiology, Faculty of Medicine, Khon Kaen University, Khon Kaen 40002, Thailand

^2^Faculty of Veterinary Medicine, Khon Kaen University, Khon Kaen 40002, Thailand

^3^Department of Anatomy, Faculty of Medicine, Khon Kaen University, Khon Kaen 40002, Thailand

^4^Research Institute for Human High Performance and Health Promotion, Khon Kaen University, Khon Kaen 40002, Thailand,

*[ppoung@kku.ac.th](mailto:ppoung@kku.ac.th)

**Supplementary information: (Figure 6a) : n = 6**

**
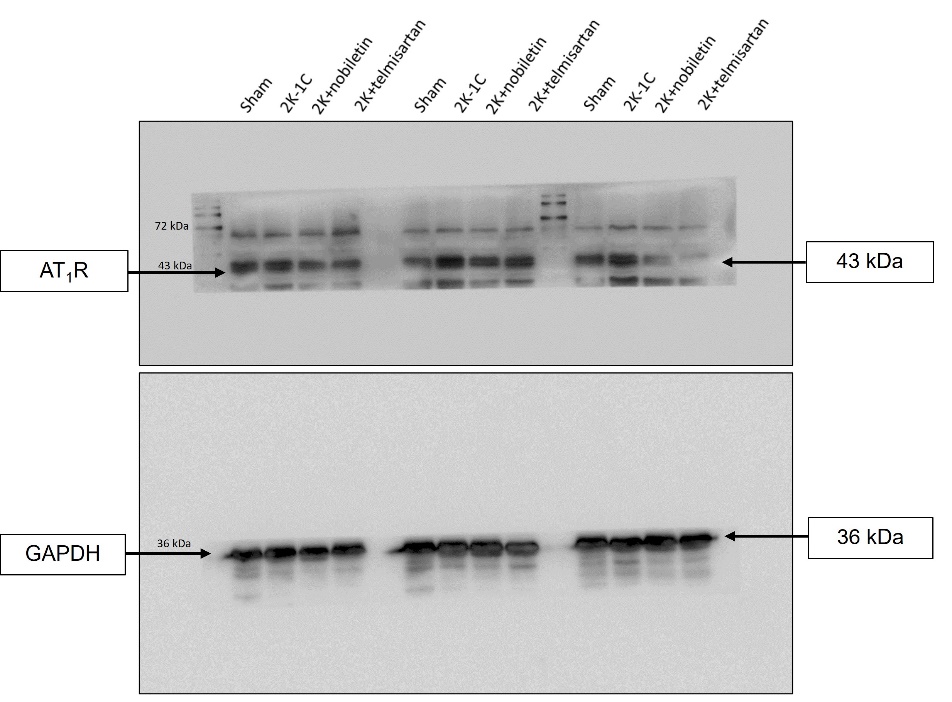
**

**n3**

**n2**

**n1**


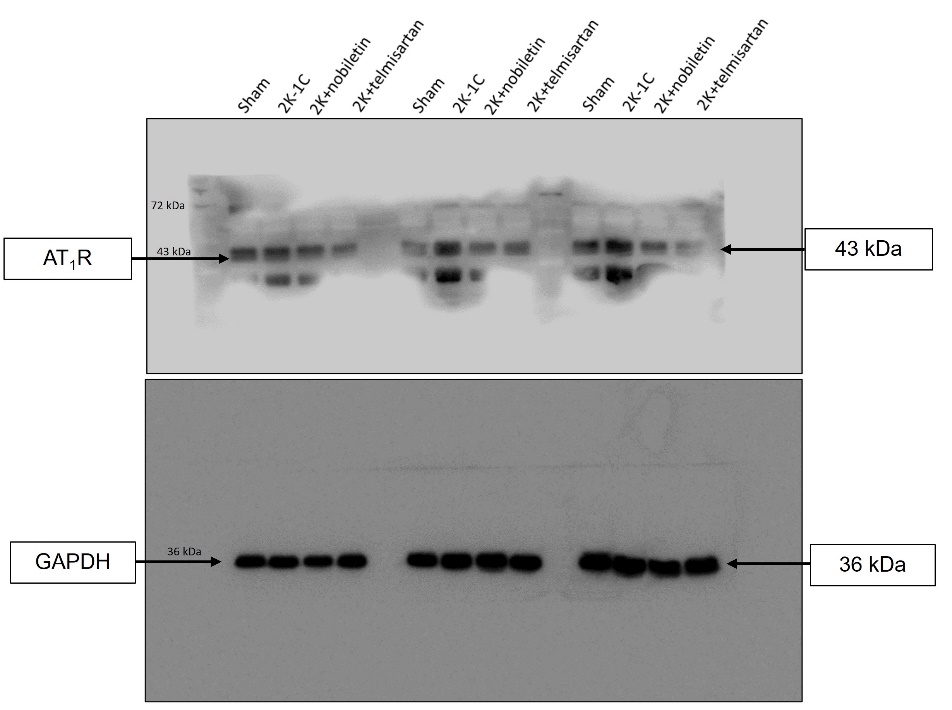


**n6**

**Representative**

**band**

**n4**

**n52**

**Supplementary information: (Figure 6b)****: n = 3**


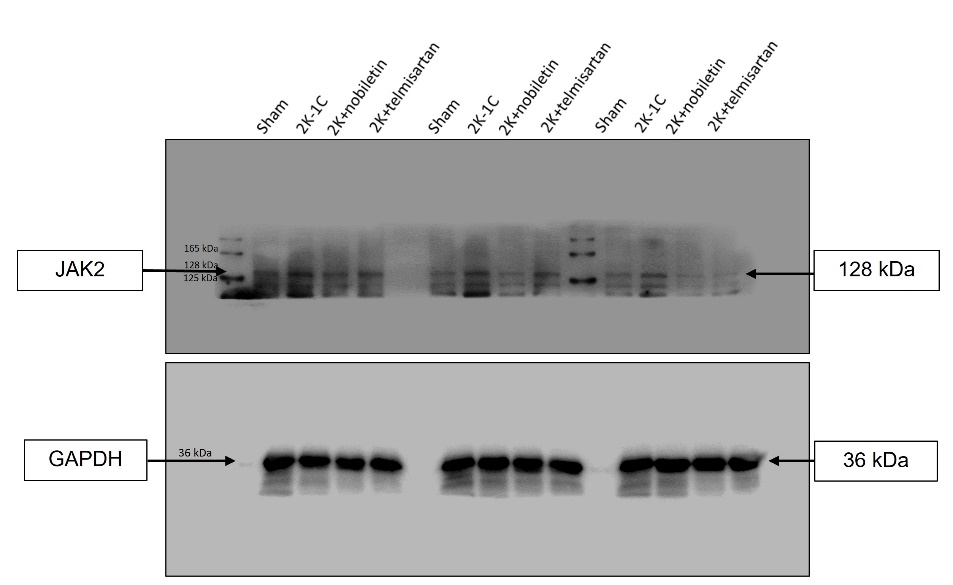


**Representative**

**band**

**n3**

**n2**

**n1**

**Supplementary information: (Figure 6c): n = 6**

**
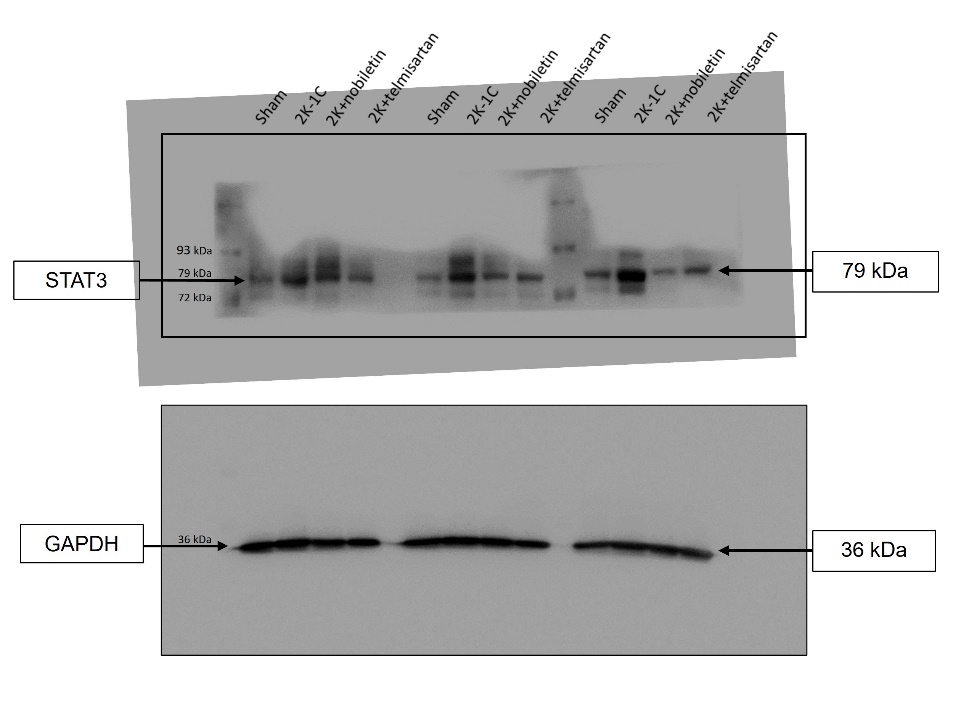
**

**n3**

**n1**

**Representative**

**band**

**n2**


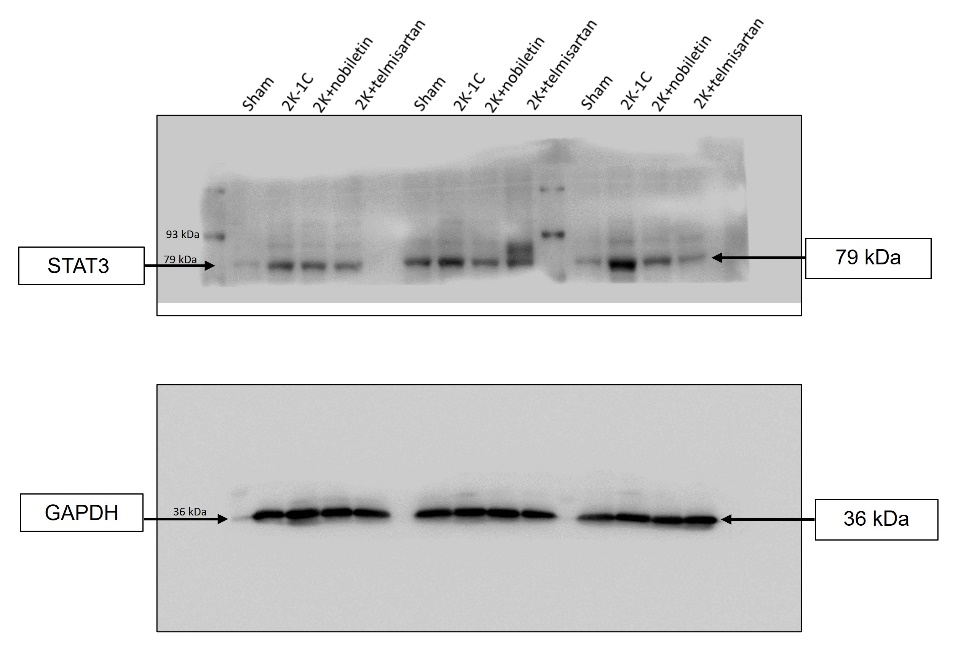


**n6**

**n4**

**n52**

**Supplementary information: (Figure 8a): n = 4**


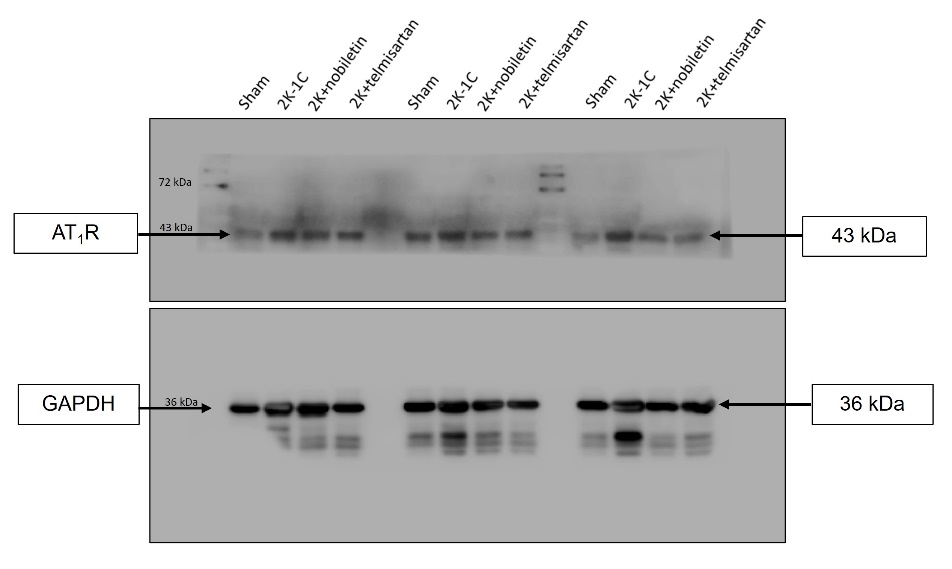


**Representative**

**band**

**n2**

**n1**

**n3**


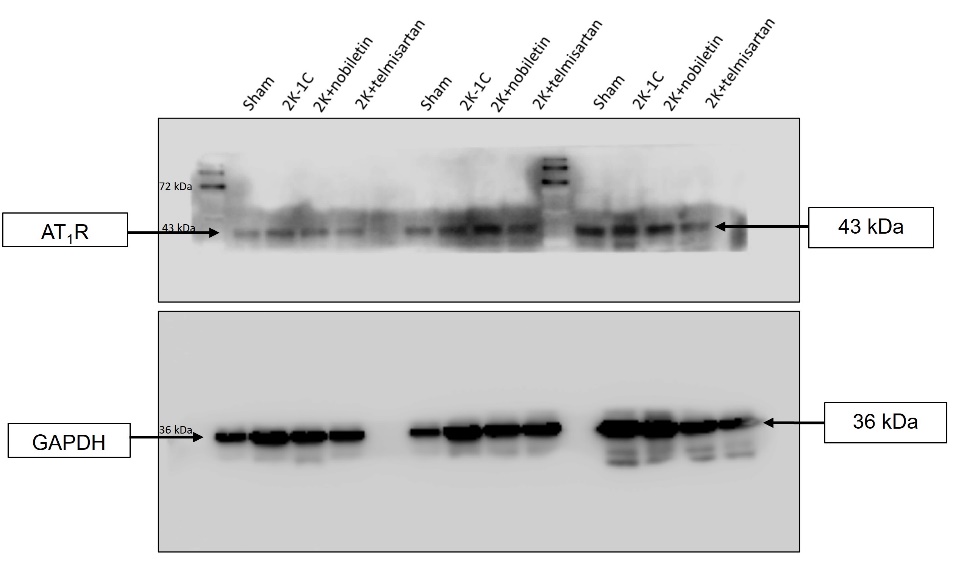


**n4**

Please note that the last two n-numbers could not be analyzed because of high background signal on the blot.

**Supplementary information: (Figure 8b): n = 5**


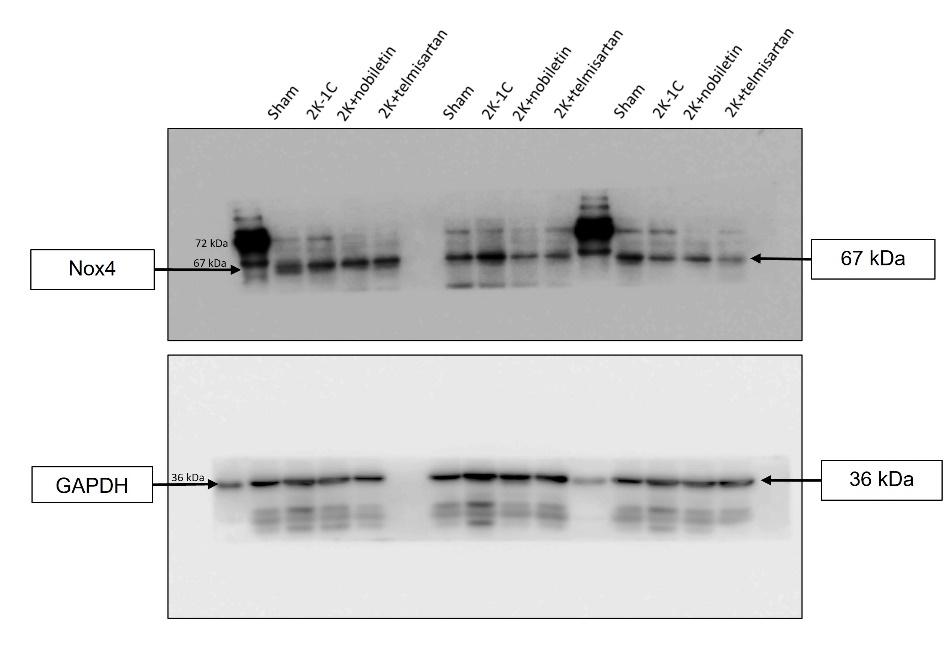


**n1**

**n3**

**n2**


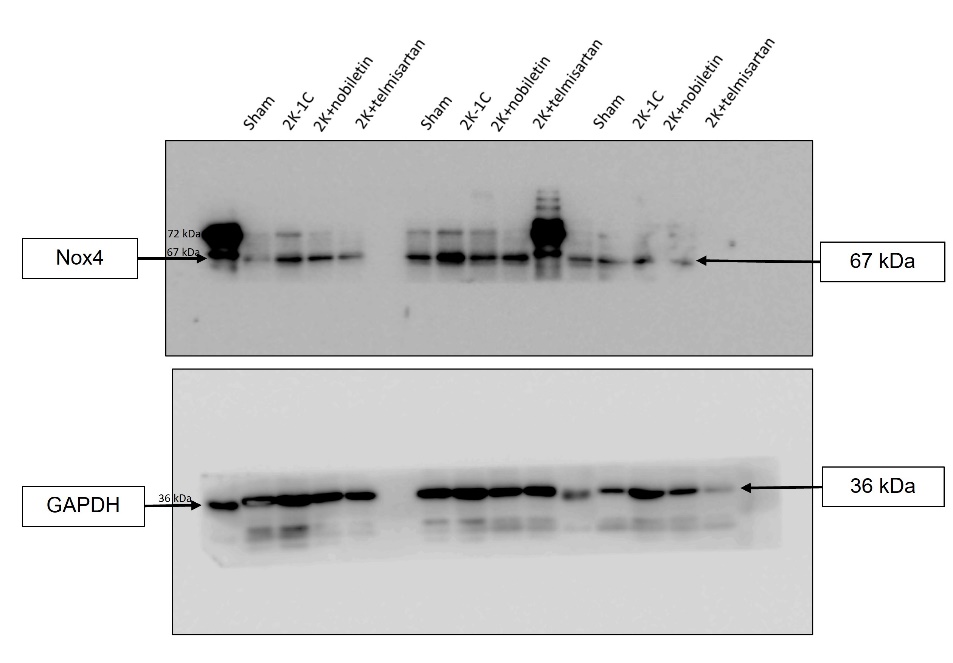


**n4**

**n52**

**Representative**

**band**

Please note that the last n-number could not be analyzed because of blotchy transfer issue.
